# Evaluation of Radiopacity of Calcium Silicate-Based and Resin-Based Materials Using Direct Digital Radiography Along With an Aluminium Step Wedge

**DOI:** 10.7759/cureus.70035

**Published:** 2024-09-23

**Authors:** Saleem Azhar, Abhinay Agarwal, Sahil Dhingra, Mohammad Salman Akhtar, Sachin Yadav, Shubham Sandesh, Vikas Singh

**Affiliations:** 1 Conservative Dentistry and Endodontics, Teerthanker Mahaveer Dental College and Research Centre, Moradabad, IND; 2 Conservative Dentistry and Endodontics, Kalka Dental College and Hospital, Meerut, IND; 3 Public Health Dentistry, Teerthanker Mahaveer Dental College and Research Centre, Moradabad, IND

**Keywords:** aluminium step wedge, clinical implication, direct digital radiography, radiopacity, root canal sealers

## Abstract

Background

Radiopacity is a critical property for root canal sealers as it allows for the assessment of the material's placement and quality within the root canal system on radiographic images. The study aimed to evaluate the radiopacity of calcium silicate-based and resin-based materials using direct digital radiography, employing an aluminium step wedge according to American National Standards Institute/International Organization for Standardization (ANSI/ISO) standard protocols for testing the radiopacity of root canal sealers. This study seeks to determine the effectiveness of these materials in meeting the required standards for clinical use.

Methodology

The materials tested were AH Plus, Apexit Plus, Biodentine and MTA Fillapex in circular disc form and radiographed alongside the aluminium step wedge using direct digital radiography to determine the radiopacity using grey-pixel values. All the materials, viz., AH Plus, Apexit Plus, Biodentine and MTA Fillapex were found satisfactorily radiopaque and met the minimum radiopacity standard, that is, minimum 3mm of aluminium recommended by ANSI/American Dental Association (ADA) Specification No. 57.

Results

AH Plus exhibited the highest radiopacity with values of 222.54 mm Al Eq (isodensity) and 220.88 mm Al Eq (densitometric), significantly surpassing Apexit Plus, Fillapex and Biodentine (p < 0.001). Apexit Plus and Fillapex showed no significant difference between them (p = 0.238), but both were significantly higher than Biodentine (p < 0.001). Biodentine had the lowest radiopacity among all the sealers tested.

Conclusion

All the tested materials met the ANSI/ADA minimum radiopacity standard, demonstrating their suitability for clinical use. The materials varied in their levels of radiopacity, demonstrating that they are adequately visible on radiographic images for effective assessment in root canal treatments.

## Introduction

Radiographs are important diagnostic tools in the field of medicine and surgery and endodontics is no exception. Endodontic treatment relies greatly on radiographs without which the success or failure of the treatment would not be evaluated [1]. Therefore, among other physical and chemical characteristics, the perfect root canal filler material should have enough radiopacity to enable differentiation from the surrounding anatomic tissues [2,3]. Thus, the radiograph is an indispensable tool for the evaluation of the outcome of endodontic treatment.

According to Grossman (1958), endodontic sealers, regardless of type, should exhibit optimal radiopacity to be distinguished from proximal anatomic structures such as tooth and bone [4]. Radiological inspection is the sole way to confirm, in a clinical setting, the quality of the root canal's final filling. For this reason, in addition to their other physical and chemical characteristics, obturation materials should be able to absorb radiation and stand out from surrounding tissues on radiographs to a significant extent [5,6].

Gutta-percha has long been utilized as a core material for sealers based on zinc oxide and eugenol. However, zinc oxide-based sealants shrink when they set and eventually dissolve, compromising the apical seal's quality and lifespan. But nowadays, newer sealers have been introduced with excellent properties that can flow easily into the dentinal tubules and minor crevices as well, when obturated along with the core material. Hence their radiopacity must fulfill the minimum requirements as suggested by International Standardization Organization (ISO) so as to visualize them on a radiograph and also to differentiate them with the core material. Since aluminium's radiopacity has been reported to be comparable to dentin's, it was used as a reference. Aluminium is utilized for the step wedge because, according to Gu et al. [7], it has a linear absorption coefficient that is comparable to enamel's, connecting aluminium's variation to that of hydroxyapatite.

A digital system in dental radiology was introduced in 1989 and since then direct digital radiography (DDR) has found its way into dental practice. DDR is a potential way to enhance the current techniques for evaluating radiopacity, according to Tagger and Katz [8]. It reduces the operator’s exposure to radiation, eliminates the need for chemical processing of radiographs and provides more consistent results; traditional film development, unless performed carefully, can produce significant variations in the final radiographs [9]. Charge-coupled devices (CCD), complementary metal oxide semiconductors (CMOS) and photo-stimulable phosphor plates (imaging plates) are a few of the sensor types that can be employed. In some studies, even radiographic images were digitized to compare the radiopacity of endodontic materials [10]. Hence it was decided to undertake the present study to determine the radiopacities of different endodontic sealers such as AH Plus, Apexit Plus, MTA Fillapex and Biodentine. DDR was employed instead of conventional or digitised radiographs. The study included in-vitro conditions to differentiate between radiopacities of three different endodontic sealers in 1mm thick sections.

## Materials and methods

Study design and setting

This study employed an experimental design conducted in a controlled laboratory setting. The primary objective was to evaluate the radiopacity of various calcium silicate-based and resin-based materials using direct digital radiography. The assessment was performed in accordance with ANSI/ISO standard protocols for testing the radiopacity of root canal sealers. An aluminium step wedge was used as a reference to measure the radiopacity of each material.

Selection Criteria

The inclusion criteria for this study required the use of commercially available root canal sealers or dental restorative materials, specifically calcium silicate-based or resin-based types, prepared according to the manufacturer's instructions in circular discs of 1 mm thickness and 4 mm diameter. Exclusion criteria included materials not classified as root canal sealers or dental restorative materials, sealers outside the specified categories, samples not prepared according to the manufacturer's guidelines and disc samples not meeting the specified dimensions.

Data sources and variables

Three root canal sealers (AH Plus, Apexit Plus, MTA Fillapex) and Biodentine were used in a total of 80 circular disc samples, which were further divided into 20 disc samples for each material. Materials were measured in equal volumes and dispensed on a glass slab (according to the manufacturer’s instructions). After complete mixing and homogenization, the materials were placed in circular wells, 1 mm deep and 4 mm in diameter. Discs of 1 mm thickness were used because previous studies have indicated that the radiopacity of sealers may vary in relation to thickness of the sample and thinner the sample, closer it is to the clinical conditions.

**Figure 1:**
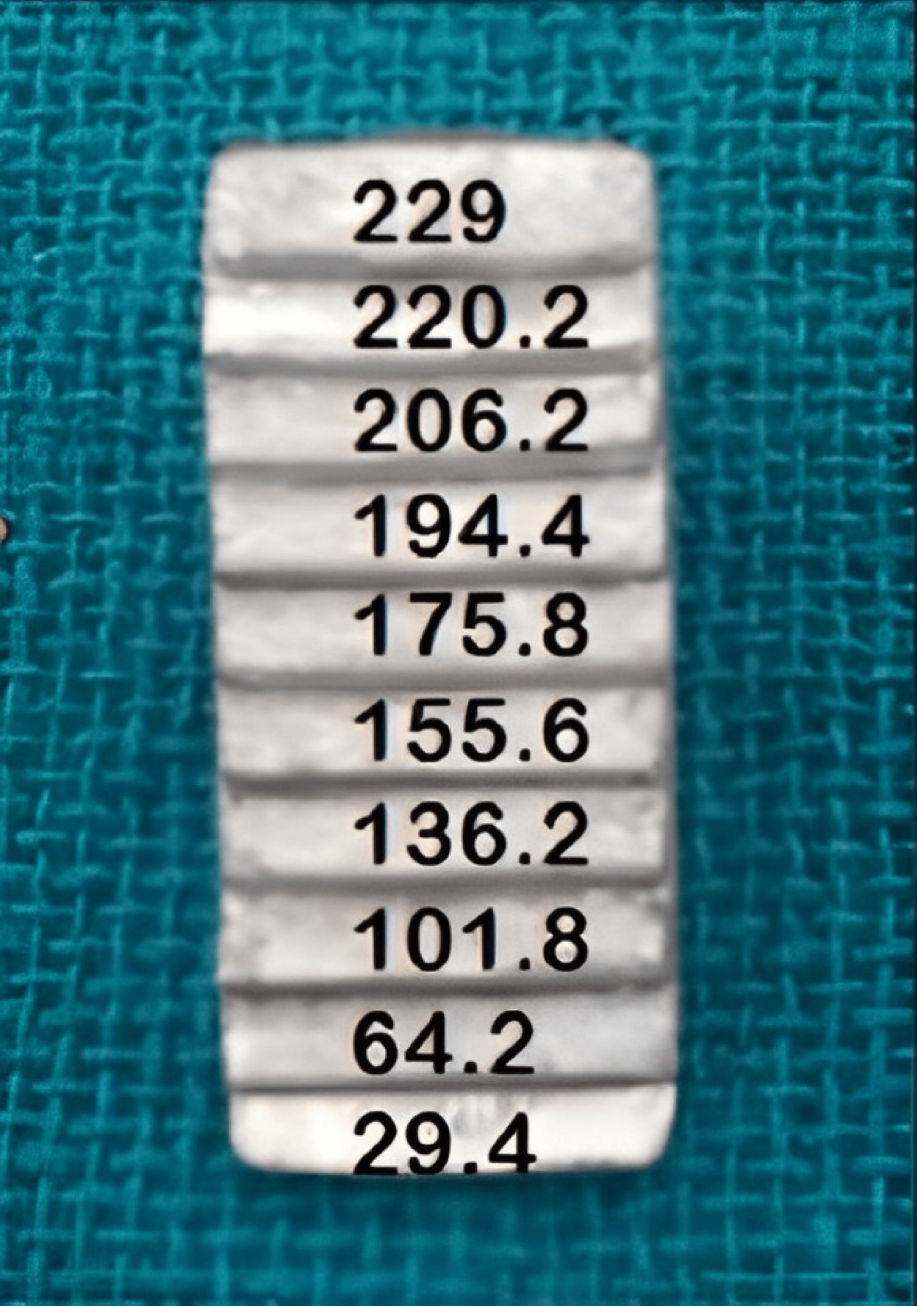
Aluminium step wedge made of Alloy 1100 and measurement of the radiopacity in grey scale value (GSV)

Materials were subjected to porosities test by radiographing them using DDR. The black dots on images of the sample indicated porosity. The materials with porosities were excluded from the study and were replaced with homogenous specimens. An aluminium step wedge made of Alloy 1100 and with 1 mm thick incremental steps was used as a standard for comparison of radiopacity of the tested materials (Figure 1).

**Figure 2:**
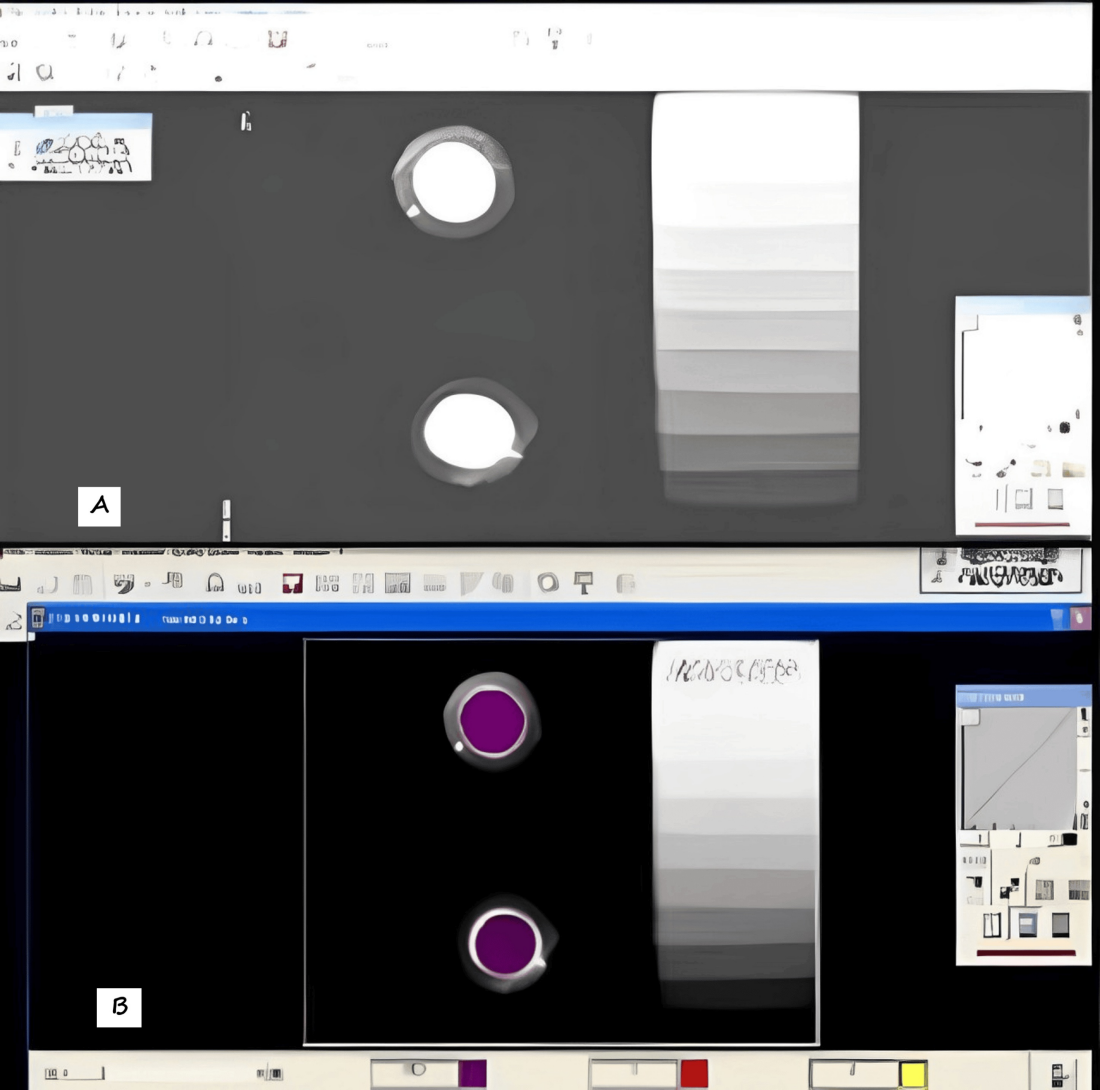
Materials were radiographed separately alongside an aluminium step wedge A: step wedge with the black and white contrast image of the material used B: step wedge with the color contrast image of the material used

Standard radiographic images were obtained using a Kodak RVG 5100 sensor (Kodak, Rochester, NY, USA) and a dental X-ray machine (KDS India, Delhi, India) operating at 70 kVp and 10 mA was used. The object-to-focus distance was 30 cm, and the exposure time 0.3 seconds. To remove the observer error and bias, two samples at a time of each material were radiographed separately alongside an aluminium step wedge that was used as a reference (Figure 2).

Digital images obtained were analysed using the Kodak’s software. The isodensity tool and the equal density regions alloying comparison between various material densities and the radiopacities of various degrees of thickness of the aluminium step wedge were identified using a densitometric analysis tool. The program represented the same radiographic density as the sample, and the matching thickness of the aluminium step wedge was found by selecting the region above the specimen with the mouse in each digital radiography picture. The aluminium step wedge and the area of the digital radiography that included the samples were treated with the grey scale tool. Only those areas that were devoid of air bubbles and other abnormalities were carefully chosen for analysis. After determining the grey scale values for each specimen, the following formula was applied to convert the values in millimeters of aluminium:

$\text{Al_equivalent (mm)} = \frac{\text{Radiopacity of the specimen} \times \text{Al thickness of step wedge (mm)}}{\text{Radiopacity of the step wedge}}$

Statistical analysis

The grey scale values of the steps of the aluminium step wedge were measured and noted using DDR. Similarly, the grey scale values of the samples were also noted. The grey scale values were then converted to its relative aluminium equivalent by the formula and noted. The observations were tabulated and IBM SPSS Statistics, version 21.0 (IBM Corp., Armonk, NY, USA) was used to apply two-way analysis of variance (ANOVA) for in-vitro study followed by post-hoc analysis using Tukey’s test.

## Results

Table 1 summarizes the mean radiopacity measurements of various root canal sealers using both the Isodensity tool and densitometric analysis. AH Plus exhibited the highest radiopacity with values of 222.54 mm Al Eq using the isodensity tool and 220.88 mm Al Eq through densitometric analysis. Apexit Plus followed with 185.71 mm Al Eq (isodensity) and 188.07 mm Al Eq (densitometric), while Fillapex measured 181.04 mm Al Eq (isodensity) and 183.32 mm Al Eq (densitometric). Biodentine showed the lowest radiopacity among the sealers, with values of 120.03 mm Al Eq (isodensity) and 114.87 mm Al Eq (densitometric). These findings provide insights into the radiopacity characteristics of each sealer, crucial for their clinical applicability in root canal treatments.

**Table 1:** Mean radiopacity of different sealers as measured by isodensity tool and densitometric analysis mm AI Eq: millimeter of aluminium equivalence

Sealer	Isodensity tool	Densitometric analysis	mm Al Eq
AH plus	222.54	220.88	9.06
Apexit plus	185.71	188.07	6.38
Fillapex	181.04	183.32	6.14
Biodentine	120.03	114.87	3.3

Table 2 presents the results of the ANOVA and post-hoc comparisons for radiopacity measurements of root canal sealers. The comparisons reveal that AH Plus exhibited significantly higher radiopacity compared to Apexit Plus, Fillapex, and Biodentine, with mean differences of 36.83, 41.50 and 102.51 mm Al Eq, respectively, all with p-values less than 0.001. In contrast, the difference in radiopacity between Apexit Plus and Fillapex was not statistically significant (mean difference of 4.24 mm Al Eq, p = 0.238). However, Apexit Plus showed a significant difference compared to Biodentine (mean difference of 65.69 mm Al Eq, p < 0.001), and Fillapex was significantly different from Biodentine (mean difference of 61.45 mm Al Eq, p < 0.001).

**Table 2:** ANOVA and post-hoc comparisons for radiopacity measurements of root canal sealers mm AI Eq: millimeter of aluminium equivalence p value < 0.05 is considered to be statistically significant

Comparison	Mean Difference (mm Al Eq)	Standard Error	t-Value	p-value
AH Plus vs. Apexit Plus	36.83	3.56	10.35	< 0.001
AH Plus vs. Fillapex	41.50	3.62	11.45	< 0.001
AH Plus vs. Biodentine	102.51	3.48	29.47	< 0.001
Apexit Plus vs. Fillapex	4.24	3.55	1.19	0.238
Apexit Plus vs. Biodentine	65.69	3.78	17.36	< 0.001
Fillapex vs. Biodentine	61.45	3.77	16.27	< 0.001

## Discussion

It is commonly known that radiopacity is a desired quality for all intraoral materials, including endodontic sealants [8]. Radiography plays a major role in dental diagnostics. Root canal sealers need to be radiopaque in order to be recognized and distinguished from the surrounding anatomical structures. The sealer should help make the root filling more radiopaque so that it can be seen on radiographs and used to assess apical ramification and lateral canal obturation [11]. The ISO states that root canal sealers should have radiopacities that are at least as high as 3 mm of aluminium [12].

In the past, the most popular technique for determining the radiopacity of root canal sealers was traditional periapical radiography. Using a labor-intensive developing and fixing solution, the radiographic film was chemically processed to produce the pictures using this approach. On the other hand, DDR is a relatively new field in radiology with a lot of potential advantages, including improved contrast, better vision, sharper pictures, and a rapid process [13]. DDR offers computer-based image processing, analysis and storage with easy retrievability for the radiographic evaluation [6]. DDR saves time and reduces the steps that might affect the radiographic quality because it does not require traditional periapical radiography film or radiographic chemical processing. It also reduces radiation exposure and provides a detailed analysis of digital images [14].

In the present study, the root canal sealers such as AH Plus, Apexit Plus, Fillapex and Biodentine were chosen for the comparison and evaluation of their radiopacity using DDR. Among these sealers, AH Plus an epoxy resin-based sealer is the most commonly used and Apexit Plus a calcium hydroxide-based sealer although in use for quite some time, not many reviews have been cited on their radiopacity. On the other hand, MTA Fillapex and Biodentine are newly introduced silicate-based materials and they too lack studies on their radiopacity. These materials were compared and evaluated for radiopacity in circular discs using DDR. Discs of 1 mm (ISO 6876/2001) were prepared using transparent plastic rings because the radiopacity of materials may vary in relation to the thickness of the sample and the thinner the sample, the closer it is to the conditions of clinical use [15]. The diameter of the samples was decreased from the ANSI/ADA specification of 10 mm to 5 mm [12,16] to allow more samples to be placed in the central part of the digital receptor. The materials selected offered a range of radiopacities due to the presence of different opacifiers in each of these materials. The ISO 6876:2001 standard establishes 3 mm Al as the minimum radiopacity for root canal sealers [12]. Aluminium step wedge was used to compare the radiopacity of root canal sealers. Aluminium of 1100 series (99% pure) was obtained from Hindalco Industries Limited (Mumbai, India) as it satisfies the requirements established by ISO standards for an aluminium step wedge. It is commonly known that pure aluminium has radiopacities that are quite similar to human dentine [12,16].

Aluminium is selected for the step wedge because, according to Bueno et al. [17], it has a linear absorption coefficient that is comparable to enamel's, connecting aluminum's fluctuation to hydroxyapatite. Some studies used a standard thickness of 2 mm for their samples and compared their images to those obtained with 4 mm of aluminium [17,18]. Other authors have used different thicknesses for their specimens, including 1 mm [12,16,19], 1.5 mm [20] and 3 mm [21] to compare with the images of their test materials. The steps in this investigation were made from a single aluminium sheet up to a thickness of 1 mm or 10 mm. The measuring procedure may be sped up using these processes, which is an extra bonus [22,23]. The radiopacity values of every substance examined in this study were higher than the lowest amount advised by the ISO standard [12,16].

Limitations of the study

This study is constrained by several factors. It evaluated only four specific root canal sealers - AH Plus, Apexit Plus, Fillapex, and Biodentine - limiting the breadth of sealers analyzed compared to those used clinically. Although direct digital radiography (DDR) was employed for its efficiency and image quality benefits, variations in equipment and settings could impact radiographic outcomes. The use of smaller diameter circular discs (4 mm instead of 10 mm) aimed to optimize sample placement but might have influenced radiopacity measurements. Future research with a broader selection of sealers and standard-sized samples could provide more comprehensive insights into radiopacity across different clinical scenarios.

## Conclusions

All the tested materials - AH Plus (9.06 mm Al), Apexit Plus (6.38 mm Al), Fillapex (6.14 mm Al), and Biodentine (3.3 mm Al) - were found to be satisfactorily radiopaque and met the minimum radiopacity standard of 3 mm of aluminium as recommended by ANSI/ADA Specification No. 57. The observed radiopacity followed the sequence: AH Plus > Apexit Plus > Fillapex > Biodentine. This indicates that while all materials are suitable for clinical use, AH Plus exhibits the highest radiopacity among the tested materials.

## References

[1] Goldman M, Simmonds S, Rush R (1989). The usefulness of dye-penetration studies reexamined. Oral Surg Oral Med Oral Pathol.

[2] Beyer-Olsen EM, Orstavik D (1981). Radiopacity of root canal sealers. Oral Surg Oral Med Oral Pathol.

[3] Katz A, Kaffe I, Littner M, Tagger M, Tamse A (1990). Densitometric measurement of radiopacity of Gutta-percha cones and root dentin. J Endod.

[4] Grossman LI (1958). An improved root canal cement. J Am Dent Assoc.

[5] Gambarini G, Testarelli L, Pongione G, Gerosa R, Gagliani M (2006). Radiographic and rheological properties of a new endodontic sealer. Aust Endod J.

[6] Imai Y, Komabayashi T (2003). Properties of a new injectable type of root canal filling resin with adhesiveness to dentin. J Endod.

[7] Gu S, Rasimick BJ, Deutsch AS, Musikant BL (2006). Radiopacity of dental materials using a digital X-ray system. Dent Mater.

[8] Tagger M, Katz A (2003). Radiopacity of endodontic sealers: development of a new method for direct measurement. J Endod.

[9] Syriopoulos K, Sanderink GC, Velders XL, van der Stelt PF (2000). Radiographic detection of approximal caries: a comparison of dental films and digital imaging systems. Dentomaxillofac Radiol.

[10] Vidotto APM, Cunha RS, Zeferino EG, Pedro Rocha DG, de Martin AS, da Silveira Bueno CE (2011). Comparison of MTA Fillapex radiopacity with five root canal sealers. Revista Sul-Brasileira de Odontologia (South Brazilian Dentistry Journal).

[11] Watts DC, McCabe JF (1999). Aluminium radiopacity standards for dentistry: an international survey. J Dent.

[12] (2001). Dental Root Canal Sealing Materials (ISO 6876:2001). https://cdn.standards.iteh.ai/samples/34965/871533aaa9a44ad79438a2229e70b1cb/ISO-6876-2001.pdf.

[13] Baksi Akdeniz BG, Eyüboglu TF, Sen BH, Erdilek N (2007). The effect of three different sealers on the radiopacity of root fillings in simulated canals. Oral Surg Oral Med Oral Pathol Oral Radiol Endod.

[14] Sabbagh J, Vreven J, Leloup G (2004). Radiopacity of resin-based materials measured in film radiographs and storage phosphor plate (Digora). Oper Dent.

[15] Wenzel A, Hintze H, Hørsted-Bindslev P (1998). Discrimination between restorative dental materials by their radiopacity measured in film radiographs and digital images. J Forensic Odontostomatol.

[16] (2000). Endodontic Sealing Materials: American National Standard/American Dental Association Specification No. 57. https://webstore.ansi.org/preview-pages/ADA/preview_ANSI+ADA+Specification+No.+57-2000.pdf.

[17] Bueno CE, Zeferino EG, Manhães LR Jr, Rocha DG, Cunha RS, De Martin AS (2009). Study of the bismuth oxide concentration required to provide Portland cement with adequate radiopacity for endodontic use. Oral Surg Oral Med Oral Pathol Oral Radiol Endod.

[18] Costa RF, Scelza MFZ, Costa AJO (2002). Radiopacity of root canal filling cements: evaluation by pixel intensity. J Bras Clin Odontol Integr.

[19] Tanomaru-Filho M, Jorge EG, Tanomaru JM, Gonçalves M (2008). Evaluation of the radiopacity of calcium hydroxide- and glass-ionomer-based root canal sealers. Int Endod J.

[20] Ungor M, Onay EO, Orucoglu H (2006). Push-out bond strengths: the Epiphany-Resilon endodontic obturation system compared with different pairings of Epiphany, Resilon, AH Plus and gutta-percha. Int Endod J.

[21] Bodrumlu E, Sumer AP, Gungor K (2007). Radiopacity of a new root canal sealer, Epiphany. Oral Surg Oral Med Oral Pathol Oral Radiol Endod.

[22] Savioli RN, Silva RG, Pécora JD (2000). Analysis of the radiopacity and pH of some root canal sealers. J Health Sci Inst.

[23] Tanomaru-Filho M, Jorge EG, Guerreiro Tanomaru JM, Gonçalves M (2007). Radiopacity evaluation of new root canal filling materials by digitalization of images. J Endod.

